# Influence of Different Surface Pretreatments on Shear Bond Strength of an Adhesive Resin Cement to Various Zirconia Ceramics

**DOI:** 10.3390/ma13030652

**Published:** 2020-02-01

**Authors:** Marco Colombo, Simone Gallo, Sara Padovan, Marco Chiesa, Claudio Poggio, Andrea Scribante

**Affiliations:** Department of Clinical, University of Pavia, Surgical, Diagnostic and Paediatric Sciences - Section of Dentistry, Piazzale Golgi 2, 27100 Pavia, Italy; marco.colombo@unipv.it (M.C.); sara.padovan02@universitadipavia.it (S.P.); marco.chiesa@unipv.it (M.C.); claudio.poggio@unipv.it (C.P.); andrea.scribante@unipv.it (A.S.)

**Keywords:** zirconia, ceramics, adhesion, CAD/CAM restorative materials, shear bond strength, surface treatment, sandblasting, air-borne particle abraded, hot etching, adhesive cement

## Abstract

The aim of this in vitro study was to assess the influence of surface pretreatment on shear bond strength (SBS) of an adhesive resin cement (G-CEM Link Force TM, GC Corporation, Tokyo, Japan) to three different yttria-stabilized tetragonal zirconia polycrystalline (Y-TZP) ceramics: (1) Copran Zirconia Monolith HT, COP; (2) Katana ML Zirconia, KAT; and (3) Metoxit Z-CAD HTL Zirconia, MET. In total, 45 cylinders (5 mm in diameter, 1 mm height) for each type of zirconia ceramic were prepared used a computer-aided design and computer-aided manufacturing (CAD/CAM) machine (software CEREC 4.2). Each type of zirconia was subdivided into three groups and each group received a different surface pretreatment; 15 samples were not conditioned as control (groups COP 1, KAT 1, MET 1), 15 samples were air-borne particle abraded with aluminum dioxide particles of 50-μm size at 0.3 MPa for 20 s (groups COP 2, KAT 2, MET 2), and 15 samples were hot-etched with a solution of hydrochloric acid and ferric chloride (groups COP 3, KAT 3, MET 3). After specimen fabrication, the adhesive cement–ceramic interface was analyzed using an SBS test. Subsequently, the adhesive remnant index (ARI) was measured. Data were submitted to statistical analysis. Air-borne particle abraded specimens showed the highest SBS values for COP and KAT groups. For MET, no significant difference was reported between air-borne particle abraded specimens and untreated controls. The lowest values were detected for acid-etched groups. A higher frequency of ARI = “1” and ARI = “2” was reported in control and air-borne particle abraded groups, whereas ARI = “3” was detected in hot-etched groups. No correlation was found between ARI score and shear bond strength. Air-borne particle abrasion is considered the best treatment for Zirconia Copran and Zirconia Katana ML, if it is followed by using dual-curing resin cement.

## 1. Introduction

In recent years, the use of aesthetic materials increased more and more in the field of dentistry. This might be the consequence of the development of new fabrication techniques and of the need to satisfy patients’ desires. Considering the current restoration systems, computer-aided design and computer-aided manufacturing (CAD/CAM) ensures the production of tooth-colored restorations of both resin and ceramic materials in a short amount of time [1].

Composite resin block materials are available for CAD/CAM technology since 2000 [2]. These blocks are manufactured by industries with standardized criteria of elevated pressure and temperature to get optimal microstructural characteristics. A CAD/CAM hybrid ceramic block was developed as a polymer-infiltrated feldspar ceramic network united to aluminum oxide [3]. During the last few years, zirconia restorations established themselves in conservative dentistry, thus allowing proper biocompatibility and great mechanical qualities [4,5,6,7,8,9]. These qualities make zirconia optimal for high-stress-bearing areas [10]. CAD/CAM systems were used in prosthodontics to realize zirconia restorations. These frameworks are made with a zirconia core layered or pressed with feldspathic porcelain. However, the outcome of restorations is based not only on mechanical and physical properties, but also on the method and pretreatment type of the substrate. The latter represents a critical variable of the whole procedure chosen by the clinic [11].

The physical and chemical pretreatments applied on the inner surfaces of the fixed prosthetic frameworks aim to optimize the cement adhesion to the restoration material. Densely sintered ceramics are based on pure alumina or zirconia. The cementation of these polycrystalline materials (considering the first to fifth generation of zirconia) was reported to be difficult [5].

Hydrofluoric acid positively enhances the surface morphology of the glass ceramics, but it cannot be used with polycrystalline materials [12]. This treatment was not effective for copings and frameworks made of a highly stable, dense crystalline structure (e.g., yttria-stabilized tetragonal zirconia polycrystalline (Y-TZP)).

The cohesion of the tooth–restoration complex with a bonding technique (through the use of resin cements) involves the establishment of a durable link even within the substructures. Several experimental pretreatments of zirconia were proposed, including air-borne particle abrasion (ABPA) with alumina in some cases followed by silanization [13], deposition of fused porcelain pearls on the surface [14], treatment with a flame produced by the ignition of butane gas [15], selective infiltration with low-fusion-point glass [16], and hot acid etching [17]. On the other hand, some authors proposed no pretreatment at all [18].

The resin cements that include in their chemical composition a functional phosphate monomer (also known as silane or 10-methacryloyloxydecyl dihydrogen phosphate (MDP)) showed reliable short and long bonding links to air abraded zirconia. This procedure is currently the most reliable method suggested for the cementation of high-density ceramic and zirconia [19,20].

Accordingly, the aim of this in vitro study was to verify the main effect of ABPA pretreatment on shear bond strength for three recently developed CAD/CAM zirconia ceramics. Furthermore, considering the resin cement used on the tested zirconia ceramics, this study aimed to assess the adhesive remnant index (ARI) score in the three different conditions tested (control, ABPA, and hot etching) and to evaluate whether a direct correlation occurs between this index and shear bond strength.

The null hypothesis of the present investigation was that there are no significant differences in bond strength values among the three tested zirconia ceramics considering different pretreatments. The second null hypothesis was that there are no significant differences in adhesive remnant index (ARI) scores among the various groups.

## 2. Materials and Methods

### 2.1. Specimen Preparation

Three different tetragonal CAD/CAM zirconia ceramics were tested in this study. The specifications of the materials used are listed in Table 1.

**Figure 1:**
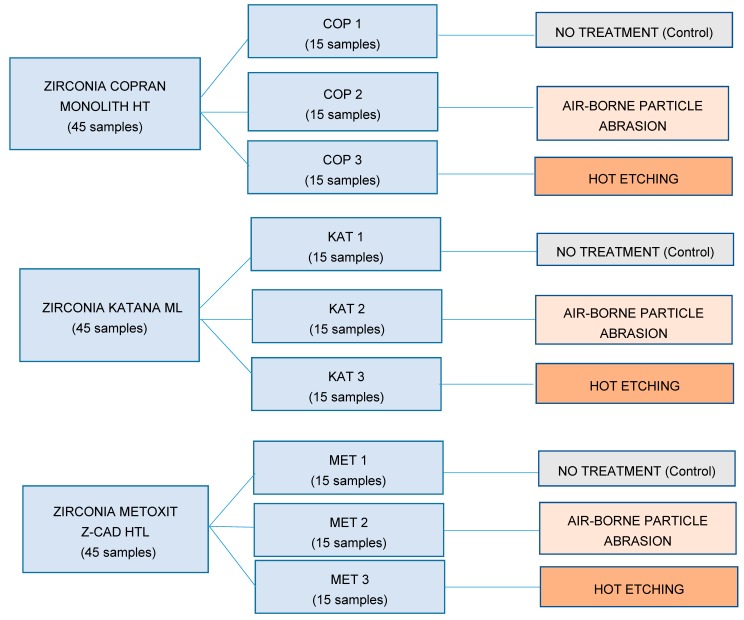
Flow chart showing how the tested materials were divided into groups and the respective treatment assigned.

In total, 45 cylinders of equal size (5 mm in diameter, 1 mm height) for each tested product were designed with CEREC software 4.2 platform. Samples of each type of zirconia were subdivided into three groups, and each one underwent a different surface treatment (no treatment, ABPA, or hot etching) as shown in the flow chart in Figure 1 and listed below.

- Groups COP 1, KAT 1, and MET 1: no treatment (control groups). The cementation surfaces remained unaltered and unchanged after the manufacturing process [21];
- Groups COP 2, KAT 2, and MET 2: ABPA (Base 1 Plus, Dentalfarm, Torino, Italia) with aluminum dioxide particles with 50-μm particle size at 0.3 MPa for 20 s at a distance of 10 mm perpendicular to the sample’s surface; subsequently, samples were rinsed with an air/water vaporizer (Minivapor.86, Effegi Brega, Sarmato, Italia) for 10 s and finally air-dried [22];
- Groups COP 3, KAT 3, and MET 3: immersion in an experimental corrosive solution of HCl–FeCl_3_ (800 mL of hydrochloric acid HCl and 5 mL of ferric chloride FeCl_3_) for 10 min at a temperature of 100 °C, subsequently washed with 90% CH_3_OH methanol and finally air-dried [17].

After the surface treatments, a liquid primer (G-Multi PRIMER, GC Corporation, Tokyo, Japan) containing methacryloyloxydecyl dihydrogen phosphate (MDP) was applied to all the samples with a microbrush to guarantee the subsequent adhesion of the resin cement to the zirconia ceramics. With an air syringe, light air flow was applied for 5 s, which allowed the solvent (ethanol) to evaporate. 

**Figure 2:**
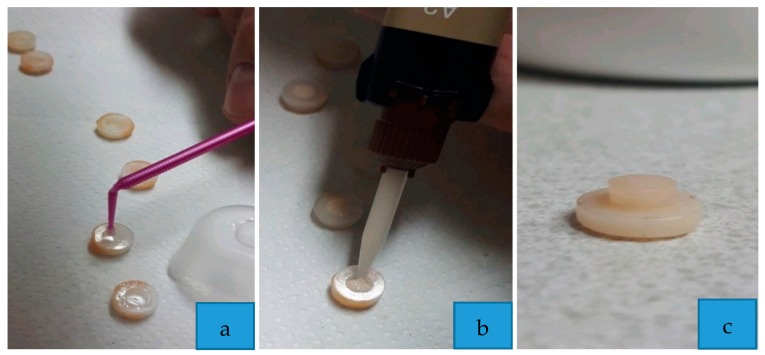
Phases of the specimen preparation: (**a**) application of primer; (**b**) application of adhesive resin cement; (**c**) specimen prepared.

In order to standardize the procedures as much as possible, the same operator applied a dual-cure adhesive luting cement (G-CEM Link Force ^TM^, GC Corporation, Tokyo, Japan), directly from the syringe inside a metallic annulus, with predetermined dimensions (external diameter 5 mm; internal diameter 3 mm; height 1 mm), which acted as a mold (Figure 2). 

The characteristics of the tested cementation system for zirconia are reported in Table 2.

After the elimination of the superficial excess cement with an Explorer 6, the samples were irradiated with light curing for 20 s using a light-emitting diode (LED) lamp (Elipar ^TM^ S10 LED Curing Light, 3M ESPE, Neuss, Germany) at a light intensity of 1200 mW/cm^2^, with a wavelength of 430–480 nm, standing with the ferrule at a distance of 1 mm from the cement [23]. The LED power was measured with the radiometer included in the instrument base. After polymerization, metallic annuli were removed, and zirconia discs were stored in saline for 7 d at a temperature of 37 °C [24].

### 2.2. Shear Bond Strength Testing

After storing, each specimen was placed in a universal testing machine (Instron Universal Testing Machine Model 3343, Instron Corporation, Norwood, MA, USA).

**Figure 3:**
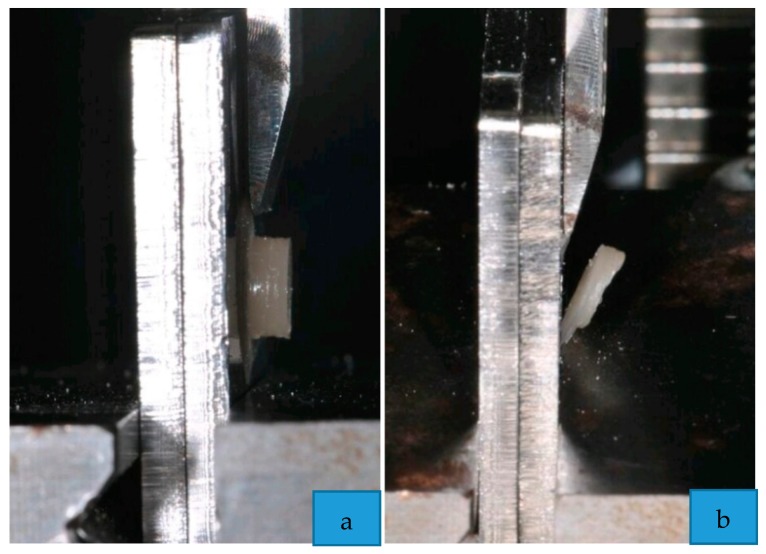
Phases of the shear bond strength testing: (**a**) application of the force; (**b**) failure of the cement.

During the test, the zirconia specimens were subjected to a force applied with a steel tip, tangentially with respect to the cementation surface, until the failure of the cement [25], as shown in Figure 3. The crosshead speed was set to 1 mm/min [26]. In order to automatically record debonding force (in newtons), the software Bluehill 2 (Instron Industrial Products, Grove City, Pennsylvania, PA, USA) was used. Data were converted into megapascals (MPa).

After detachment, the external surface of each zirconia’s sample, where adhesive was applied, was examined using an optical microscope to determine the proportion of residual adhesive using the adhesive remnant index (ARI) that evaluated the zirconia–cement interface. The ARI scale has a range between 0 and 3: (0) all the cement remained on the zirconia surface; (1) more than half of the cement remained on the zirconia surface; (2) less than half of the cement remained on the zirconia surface; (3) no adhesive remained on the zirconia surface [27].

### 2.3. Statistical Analysis

Statistical analysis was performed with R software (R version 3.1.3, R Development Core Team, R Foundation for Statistical Computing, Wien, Austria). Descriptive statistics, including the mean and the standard deviation, were calculated for each group. The normality of the data was calculated with the Kolmogorov–Smirnov test. Analysis of variance (ANOVA) was applied to establish whether significant differences in debond strength values existed among the groups. Tukey’s test was used post hoc. Significance for all statistical tests was set at *p* < 0.05. 

Finally, a frequency analysis of the ARI scores was carried out to assess the presence of differences between the residual adhesive indices of the different groups tested by means of the chi-square test. The level of significance for all tests was set at *p* < 0.05.

## 3. Results 

Descriptive statistics are presented in Table 3. 

**Figure 4:**
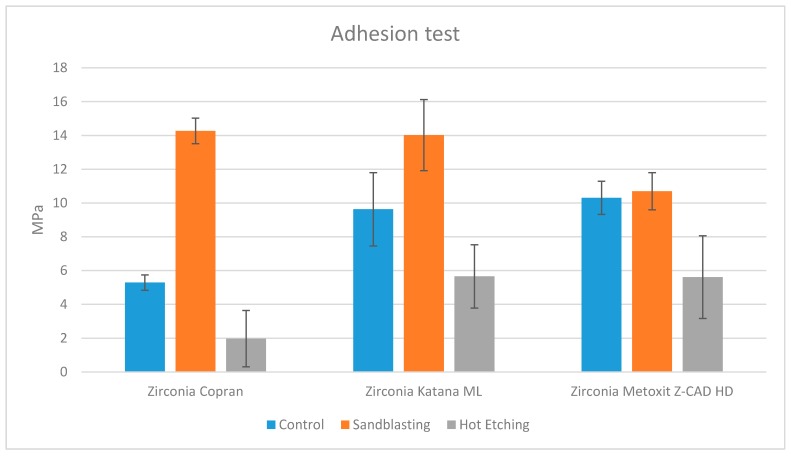
Shear forces of the groups tested.

ANOVA showed the presence of significant differences among the various groups (*p* < 0.0001). According to post hoc Tukey testing, group COP 2 (Zirconia Copran Monolith HT air-particle abraded) and group KAT 2 (Zirconia Katana ML air-particle abraded) showed significantly higher bond strengths (*p* < 0.05), as represented in Figure 4. No significant difference was detected between them (*p* > 0.05). Groups KAT 1, MET 1, and MET 2 showed detachment forces not different from each other (*p* > 0.05) and significantly lower than groups COP 2 and KAT 2 (*p* < 0.05) but significantly higher than the other groups (*p* < 0.05). Groups COP 1, KAT 3, and MET 3 showed detachment forces that were not different from each other (*p* > 0.05) and significantly higher than group COP 3 (*p* < 0.05) but significantly lower than the other groups (*p* < 0.05). The lowest detachment forces were detected by group COP 3 (*p* < 0.05). Analyzing the effect of pretreatment, significantly higher forces were found in the groups (COP 2, KAT 2, MET 2) subjected to ABPA (*p* < 0.05), whereas significantly lower values were detected in the groups (COP 3, KAT 3, MET 3) subjected to pretreatment with hydrochloric acid and ferric chloride (*p* < 0.05). Intermediate values were detected in the control groups without pretreatment (COP 1, KAT 1, MET 1).

ARI scores are presented in Table 4. The chi-square test showed that the control and air-particle abraded groups showed a higher frequency of ARI = “1” and ARI = “2” scores, while the groups subjected to pretreatment with hydrochloric acid and ferric chloride showed a higher frequency of ARI scores = “3” (*p* < 0.05).

**Figure 5:**
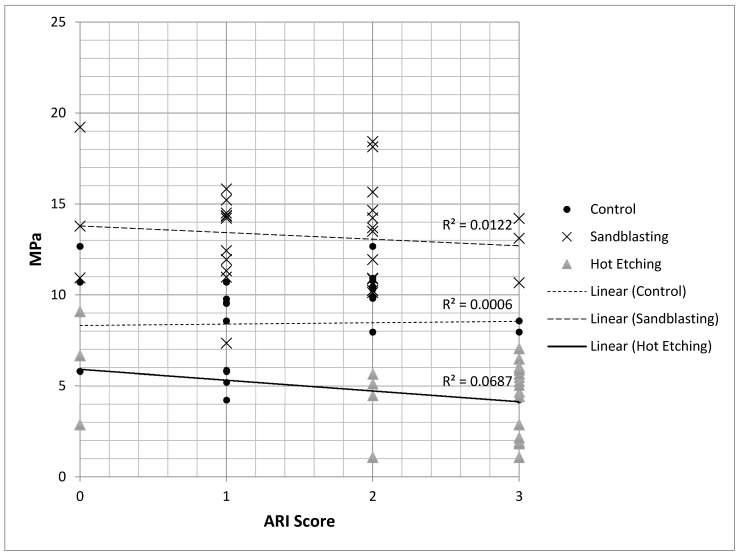
Correlation between adhesion strength (MPa) and adhesive remnant index (ARI score). No correlation between the two variables was recorded in the three different conditions tested (control, ABPA, and hot etching).

As shown in Figure 5, no significant correlation (*R^2^* < 0.0687) was recorded between adhesion strength (MPa) and adhesive remnant index (ARI Score) in the three different conditions tested (control, ABPA, and hot etching).

## 4. Discussion

Zirconia is an innovative material in the panorama of modern dentistry. It combines excellent aesthetic characteristics with many exceptional mechanical properties that make it suitable for the production of restorations in the posterior areas of the oral cavity [28].

In recent years, zirconia-based ceramics became the primary choice for the production of conventional fixed prostheses thanks to their aesthetic performance, their excellent mechanical properties, and their biocompatibility with the oral cavity [29]. The main problem that prevented the use of metal-free restorations is the ceramic having some micro-defects in its structure that over time tend to widen and eventually lead to fracture and failure of the prosthesis. This is true for almost all ceramic materials, but not for zirconia, which can overcome this problem by transforming the tetragonal zirconia grains into monoclinic zirconia grains (t → m), thus allowing the material to significantly increase its strength [30].

Conditio sine qua non to achieve complete clinical success and longevity of the restoration is the realization of a strong bond between the resin-based cement and the zirconium oxide restoration [12]. This is possible through the formation of both chemical bonds and micromechanical connections: the latter allow an increase in the surface roughness of the restoration, while the former allow obtaining a chemical adhesion between the two substrates [31].

Over the years, the pretreatment on zirconium oxide-based surfaces was extensively studied in order to make the bond with cement more effective and lasting. Due to the nature of the type of material, this research is very complex and still unfinished. Some authors showed that ABPA reduces the mechanical properties of zirconia [32], creating microfractures and alterations at the surface of the restoration. On the other hand, other research stated that the use of resinous cements containing specific phosphate monomers (10-MDP), combined with the ABPA procedure, seems to produce particularly effective results in guaranteeing stability to the restoration, as well as excellent retention, and minimizing the marginal gaps, thus avoiding the danger of infiltration and, therefore, the appearance of secondary caries [33]. Some authors used a solution based on hydrochloric acid heated to 100 °C to etch the metal arms of Maryland bridges [17], and a similar pretreatment was also proposed for zirconia ceramics. Hydrochloric acid is a highly corrosive substance capable of attacking metals such as iron, steel, and lead. However, the presence of hydrochloric acid alone is not capable of causing corrosion at the level of the zirconium surface, but the presence of oxidizing impurities such as the ferric ion (Fe^3+^) or the copper ion (Cu^2+^) leads to the breaking of the protective film of the zirconium oxide, allowing local corrosion and the creation of micro porosity. Hot etching, however, presents some limits; the vapors of the boiling solution must be aspirated by means of large hoods, and the manipulation of these caustic substances by the technician in the laboratory could constitute another important obstacle to the practical realization of this pre-treatment [34]. The acid etching of the restoration negatively influences the interaction between cement and zirconia. The ineffectiveness of this treatment method on zirconia is probably related to the physical properties of the material itself and to its high crystalline content [35]. Currently, ABPA is considered the best treatment for zirconia, which is followed by using auto-curing or dual-curing resin cements [36]. The efficacy of this technique was confirmed by both in vitro and clinical studies, reporting that ABPA with alumina particles at a moderate pressure followed by the application of an MDP-containing primer and of a composite resin luting cement provides long-term adhesion to zirconia ceramics [37,38,39].

According to results reported in literature, one of the purposes of this in vitro study was to confirm the main efficacy of ABPA, compared to hot etching and no pretreatment, on the shear bond strength of an adhesive resin cement to three recent zirconia ceramics. The first null hypothesis of the present report was partially rejected. As demonstrated, the adhesion values of the air-borne particle abraded zirconia were superior (COP 2—Zirconia Copran Monolith HT; KAT 2—Zirconia Katana ML) or equal (MET 2—Zirconia Metoxit Z-CAD HTL) to the untreated zirconia samples (groups COP 1, KAT 1, and MET 1, respectively) and the etched samples (groups COP 3, KAT 3, and MET 3 respectively).

It is of interest to point out that, only for Metoxit Z-CAD HTL, ABPA pretreatment caused an increase in strength bond values not statistically significant if compared to controls. Considering that values for untreated Metoxit Z-CAD HTL were even higher than those of Copran Monolith HT and Katana ML in the same condition, we can assert that the cementation system used in this report was effective as well. Therefore, the outcome regarding Metoxit Z-CAD HTL might be explained considering the chemical composition of this material or, more likely, its surface structure, which might be less responsive to ABPA. 

With regard to the ABPA procedure, many studies in the literature described the elimination of abraded zirconia particles before proceeding with cementation. This might be performed by rinsing the samples with water or air/water spray [40,41] or through an ultrasonic bath containing distilled water or ethanol [22,41,42]. On the contrary, no rinsing of air-borne particle abraded zirconia was described by authors, taking into account a risk of alteration of the freshly air-abraded surface with a reduction of the bonding efficacy, despite other studies clearly showing that the cleaning method has little or no effect [43]. In the present report, rinsing was conducted for all the samples through an air/water vaporizer; this procedure seems to have caused no negative effects considering that air-borne particle abraded zirconia ceramics reported debonding forces significantly higher than untreated or hot-etched ones, except for Metoxit Z-CAD HTL, as previously discussed.

The second purpose of this study was to assess the adhesive remnant index (ARI) score in the three different conditions tested (control, ABPA, and hot etching) for each material. The adhesive remnant index (ARI) is a method of defining the adhesion failure site [44]. It is widely reported in orthodontics, even if some authors showed its use in conservative dentistry [45]. The second null hypothesis of the present report was partially rejected. The chi-square test showed that the control groups and the air-borne particle abraded groups showed greater residual adhesive indices (ARI) of ”1” and ”2”, whereas specimens pretreated with hydrochloric acid and ferric chloride showed a higher prevalence of ARI = ”3”. However, a direct correlation between adhesion strength and adhesive remnant index score was not demonstrated.

Adhesion strength tests were extensively documented, and they were found to be useful to test new materials before clinical use [46]. The limitations of the present report are related to the fact that a limited number of zirconia samples were tested and that an experimental laboratory report cannot simulate intraoral conditions. Moreover, the materials were tested one week after bonding; thus, other storage times and temperatures can have a significant influence on the adhesion properties. Finally, the hydrolytic durability of the bonded specimens was not tested. Therefore, a direct extrapolation of the results of the present in vitro investigation to clinical practice must be carefully conducted. Further in vivo studies and randomized controlled trials (RCTs) are required in order to confirm the results of the present in vitro report.

## 5. Conclusions

The aim of this in vitro study was to assess the influence of surface pretreatment on shear bond strength of an adhesive resin cement (G-CEM Link Force TM, GC Corporation, Tokyo, Japan) to three different zirconia ceramics. Adhesion values of the air-borne particle abraded Zirconia Copran and Zirconia Katana ML were superior to the untreated zirconia, whereas they were equal considering air-borne particle abraded Zirconia Metoxit Z-CAD HTL. On the contrary, the hot-etched samples showed the lowest values. 

Moreover, both the untreated and the air-borne particle abraded zirconia ceramics showed a higher frequency of ARI = 1 and ARI = 2, whereas hot-etched zirconia ceramics showed a higher frequency of ARI = 3. However, no direct correlation was found between ARI score and shear bond strength.

## Figures and Tables

**Table 1:** Characteristics of the zirconia ceramics tested in the present report, according to data published by manufacturers and literature sources.

Product	Chemical Composition (wt.%) *	Technical Data *	Manufacturer (Lot No.)
Copran Zirconia Monolith HT	Zirconium dioxide (balance), yttrium oxide (5.15–5.55), aluminium oxide (0.03–0.07), iron hydroxide (0–0.01), other (0–0.02) ^a^	Density (g/cm^3^): 6.09 ^a^ Flexural strength (MPa): 1100 ^a^ Translucency (%): 20 ^b^	Whitepeaks Dental Solutions GmbH & Co. KG, Wesel, Germany (IM1024A2)
Katana ML Zirconia	Zirconium dioxide (86.21), yttrium oxide (10.95), aluminium oxide (0.16), hafnium dioxide (2.41) ^b^	Density (g/cm^3^): 6.00 ^a^ Flexural Strength (MPa): 1125 ^a^ Fracture Strength (MPa√m): 5 ^a^ Translucency (%): 31 ^a^ Grain size (μm): 0.6 ^b^	Kuraray Noritake Dental Inc., Tokyo, Japan (DDZHM)
Metoxit Z-CAD HTL Zirconia	Zirconium dioxide (92.72), yttrium oxide (5.35), hafnium dioxide (1.88), alluminium oxide (0.05), other (≤ 0.05) ^a^	Density (g/cm^3^): 6.08 ^a^ Flexural Strength (MPa): > 1100 ^a^ Translucency (%): 41 ^a^ Grain size (μm): < 0.4 ^a^	Metoxit AG, Thayngen Switzerland (409800)

* Data according to: ^a^ Manufacturers; ^b^ literature sources.

**Table 2:** Characteristics of the cementation system tested in this study, according to data published by manufacturers.

Product	Description	Chemical Composition *	Manufacturer (Lot No.)
**G-CEM Link** **Force ^TM^**	Dual-cure adhesive luting cement with barium fillers (size of 300 nm, 62 vol.% rate)	Paste A: bis-GMA, urethanedimethacrylate, dimethacrylate, barium glass, initiator, pigments. Paste B: bis-MEPP, urethanedimethacrylate, dimethacrylate, barium glass, initiator.	GC Corporation, Tokyo, Japan (1803271)
**G-Multi PRIMER**	Universal primer for adhesive cementation	Ethanol, MDP, MDTP, silane, methacrylate monomer	GC Corporation, Tokyo, Japan (1806051)

* Legend of the chemical components: Bis-GMA: bisphenol A-glycidyl methacrylate; Bis-MEPP: bisphenol-A ethoxylate dimethacrylate; MDP: methacryloyloxydecyl dihydrogen phosphate; MDTP: methacryloyloxydecyl dihydrogen thiophosphate; Silane: γ-methacryloxypropyl trimethoxysilane.

**Table 3:** Descriptive statistics (MPa) of the different groups. SD: standard deviation; Min: minimum value; Mdn: median; Max: maximum value. Superscript letters (a, b, c, and d) are used to indicate statistical results: different letters indicate the presence of significant differences in shear bond strength among the groups (significance was set at *p* < 0.05).

Group	Zirconia Type	Condition	Mean	SD	Min	Median	Max
COP 1	Copran Monolith HT	Control	5.27 ^a^	0.75	4.23	5.79	5.84
COP 2	Copran Monolith HT	Air-borne particle abrasion (APBA)	14.33 ^b^	2.35	10.12	14.34	19.22
COP 3	Copran Monolith HT	Hot etching	1.95 ^c^	0.85	1.07	1.87	2.86
KAT 1	Katana ML	Control	9.64 ^d^	0.88	8.56	9.67	10.70
KAT 2	Katana ML	Air-borne particle abrasion	14.29 ^b^	2.56	10.66	14.01	18.43
KAT 3	Katana ML	Hot etching	5.71 ^a^	0.86	4.42	5.66	7.04
MET 1	Metoxit Z-CAD HTL	Control	10.34 ^d^	1.61	7.95	10.38	12.66
MET 2	Metoxit Z-CAD HTL	Air-borne particle abrasion	11.07 ^d^	1.89	7.34	10.92	14.20
MET 3	Metoxit Z-CAD HTL	Hot etching	5.53 ^a^	1.70	2.15	5.66	9.08

**Table 4:** Percentage frequencies of distribution of ARI scores.

Group	Zirconia Type	Condition	ARI = 0	ARI = 1	ARI = 2	ARI = 3
COP 1	Copran Monolith HT	Control	10.00	80.00	0.00	10.00
COP 2	Copran Monolith HT	ABPA	10.00	70.00	10.00	10.00
COP 3	Copran Monolith HT	Hot etching	10.00	0.00	10.00	80.00
KAT 1	Katana ML	Control	20.00	60.00	10.00	10.00
KAT 2	Katana ML	ABPA	10.00	10.00	70.00	10.00
KAT 3	Katana ML	Hot etching	10.00	0.00	10.00	80.00
MET 1	Metoxit Z-CAD HTL	Control	10.00	0.00	80.00	10.00
MET 2	Metoxit Z-CAD HTL	ABPA	10.00	30.00	50.00	10.00
MET 3	Metoxit Z-CAD HTL	Hot etching	10.00	0.00	20.00	70.00
